# Supplementary material for: Resolving the full spectrum of human genome variation using Linked-Reads
Source: Genome Res. 2019 Apr;29(4):635–45. doi: 10.1101/gr.234443.118 (PMC6442396; doi:10.1101/gr.234443.118)
Supplement: Supplemental Material [file supp_gr.234443.118_Supplemental_longranger-2.2.zip › longranger-2.2.tar.bz2!/lr2.2-public/lib/stan/cmdstan/stan/doc/api/html/struct_eigen_1_1_num_traits_3_01stan_1_1agrad_1_1var_01_4.html]

Stan: Eigen::NumTraits< stan::agrad::var > Struct Template Reference


|  |  |
| --- | --- |
|  | Stan  2.5.0  probability, sampling & optimization |


- Main Page
- Related Pages
- Namespaces
- Classes
- Files

- Class List
- Class Index
- Class Hierarchy
- Class Members

All Classes Namespaces Files Functions Variables Typedefs Enumerations Enumerator Friends Macros Pages

- Eigen
- NumTraits< stan::agrad::var >

Public Types |
Static Public Member Functions |
List of all members

Eigen::NumTraits< stan::agrad::var > Struct Template Reference

Numerical traits template override for Eigen for automatic gradient variables.
More...

`#include <Eigen_NumTraits.hpp>`

|  |  |
| --- | --- |
| Public Types | |
| enum | {     IsInteger = 0, IsSigned = 1, IsComplex = 0, RequireInitialization = 0,     ReadCost = 1, AddCost = 1, MulCost = 1, HasFloatingPoint = 1   } |
|  | Properties for automatic differentiation variables read by Eigen matrix library. More... |
|  | |
| typedef stan::agrad::var | Real |
|  | Real-valued variables. More... |
|  | |
| typedef stan::agrad::var | NonInteger |
|  | Non-integer valued variables. More... |
|  | |
| typedef stan::agrad::var | Nested |
|  | Nested variables. More... |
|  | |

|  |  |
| --- | --- |
| Static Public Member Functions | |
| static Real | epsilon () |
|  | Return standard library's epsilon for double-precision floating point, `std::numeric_limits<double>::epsilon()`. More... |
|  | |
| static Real | dummy\_precision () |
|  | Return dummy precision. More... |
|  | |
| static Real | highest () |
|  | Return standard library's highest for double-precision floating point, `std::numeric_limits<double>max()`. More... |
|  | |
| static Real | lowest () |
|  | Return standard library's lowest for double-precision floating point, `&#45;std::numeric_limits<double>max()`. More... |
|  | |

## Detailed Description

### template<> struct Eigen::NumTraits< stan::agrad::var >

Numerical traits template override for Eigen for automatic gradient variables.

Definition at line 65 of file Eigen\_NumTraits.hpp.

## Member Typedef Documentation

typedef stan::agrad::var Eigen::NumTraits< stan::agrad::var >::Nested

Nested variables.

Required for numerical traits.

Definition at line 85 of file Eigen\_NumTraits.hpp.

typedef stan::agrad::var Eigen::NumTraits< stan::agrad::var >::NonInteger

Non-integer valued variables.

Required for numerical traits.

Definition at line 78 of file Eigen\_NumTraits.hpp.

typedef stan::agrad::var Eigen::NumTraits< stan::agrad::var >::Real

Real-valued variables.

Required for numerical traits.

Definition at line 71 of file Eigen\_NumTraits.hpp.

## Member Enumeration Documentation

anonymous enum

Properties for automatic differentiation variables read by Eigen matrix library.

| Enumerator | |
| --- | --- |
| *IsInteger* |  |
| *IsSigned* |  |
| *IsComplex* |  |
| *RequireInitialization* |  |
| *ReadCost* |  |
| *AddCost* |  |
| *MulCost* |  |
| *HasFloatingPoint* |  |

Definition at line 128 of file Eigen\_NumTraits.hpp.

## Member Function Documentation

|  |  |  |  |  |
| --- | --- | --- | --- | --- |
| static Real Eigen::NumTraits< stan::agrad::var >::dummy\_precision | ( |  | ) |  |

inlinestatic

Return dummy precision.

Definition at line 100 of file Eigen\_NumTraits.hpp.

|  |  |  |  |  |
| --- | --- | --- | --- | --- |
| static Real Eigen::NumTraits< stan::agrad::var >::epsilon | ( |  | ) |  |

inlinestatic

Return standard library's epsilon for double-precision floating point, `std::numeric_limits<double>::epsilon()`.

Returns
:   Same epsilon as a `double`.

Definition at line 93 of file Eigen\_NumTraits.hpp.

|  |  |  |  |  |
| --- | --- | --- | --- | --- |
| static Real Eigen::NumTraits< stan::agrad::var >::highest | ( |  | ) |  |

inlinestatic

Return standard library's highest for double-precision floating point, `std::numeric_limits<double>max()`.

Returns
:   Same highest value as a `double`.

Definition at line 110 of file Eigen\_NumTraits.hpp.

|  |  |  |  |  |
| --- | --- | --- | --- | --- |
| static Real Eigen::NumTraits< stan::agrad::var >::lowest | ( |  | ) |  |

inlinestatic

Return standard library's lowest for double-precision floating point, `&#45;std::numeric_limits<double>max()`.

Returns
:   Same lowest value as a `double`.

Definition at line 120 of file Eigen\_NumTraits.hpp.

---

The documentation for this struct was generated from the following file:

- src/stan/agrad/rev/matrix/Eigen\_NumTraits.hpp


---

[ Stan Home Page ]

*© 2011–2014,
Stan Development Team.*
